# Paget’s disease of bone: when and why to refer to specialist care

**DOI:** 10.3399/bjgp20X713369

**Published:** 2020-10-30

**Authors:** Catherine Nairn, Stuart H Ralston

**Affiliations:** St Triduana’s Medical Practice, Edinburgh.; Centre for Genomic and Experimental Medicine, MRC Institute of Genetics and Molecular Medicine, University of Edinburgh, Western General Hospital, Edinburgh.

## INTRODUCTION

Paget’s disease of bone (PDB) is characterised by increased but disorganised bone remodelling, leading to various complications including pain, deformity, and fracture. It is a relatively uncommon condition but important to recognise as it is a potentially treatable cause of bone pain. This article provides guidance to primary care teams on the diagnosis and management of patients with PDB based on a recent clinical guideline.^1^

## PATHOGENESIS AND EPIDEMIOLOGY

PDB is caused by increased osteoclast activity that is coupled with increased, but disorganised, bone formation, causing the affected bones to become deformed and more susceptible to fracture. Paget’s disease is rare below the age of 50 but becomes progressively more common thereafter and males are affected more often than females (1.4:1).^2^ Paget’s disease has a strong genetic component and particularly targets people of British descent.^2^ In some families, it is inherited as an autosomal dominant trait, most commonly because of mutations in the *SQSTM1* gene whereas, in others, there is familial clustering with no clear pattern of inheritance.^3^ Emerging evidence indicates that susceptibility to PDB is accounted for, in part, by inheritance of variants in genes that cause upregulation of osteoclast activity. Environmental factors also contribute, however, as evidenced by a reduction in prevalence and severity of the disease over the past 5 0 years. ^2^ Suggested environmental triggers include viral infections and calcium and vitamin deficiency during skeletal growth and skeletal trauma. The viral hypothesis has been studied experimentally but the data are conflicting and the role of other environmental factors has not been rigorously studied.^1^

## WHEN SHOULD PDB BE SUSPECTED?

The most common presentation is with bone pain but PDB can also present with bone deformity, deafness, or pathological fractures.^4^ In about one-fifth of patients, it is discovered as an incidental finding with an isolated elevation in alkaline phosphatase level (ALP) in a patient with otherwise normal liver function tests or by an abnormality on skeletal imaging. Rarely, PDB may present with osteosarcoma (0.3% of patients), which usually presents with a local increase in swelling and pain at an affected site.

## WHAT SHOULD A GP DO ABOUT A PATIENT SUSPECTED OF HAVING PDB?

**Figure 1:**
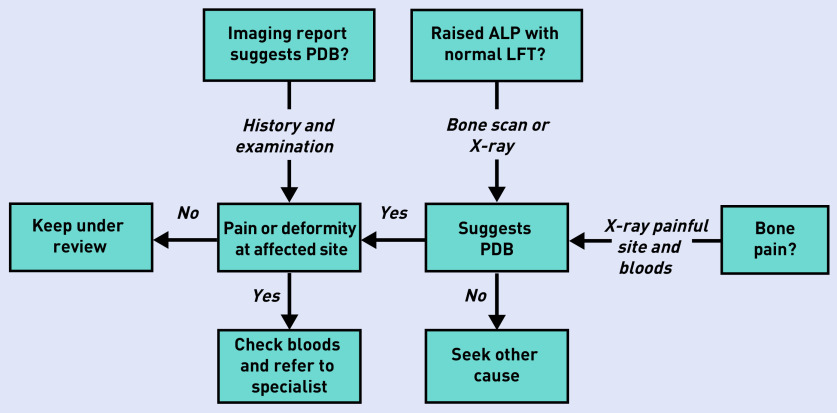
**Diagnosis and referral of patients suspected of having PDB. ALP = alkaline phosphatase level. LFT = liver function test. PDB = Paget’s disease of bone.**

A suggested pathway for diagnosis and referral is shown in Figure 1. Patients with an isolated elevation in ALP should be considered for radionuclide bone scan imaging as this is the most sensitive way of detecting PDB.^1^ If bone scans are not readily available, then plain X-rays of the abdomen (including the ribs and femoral heads), both tibias, the skull, and facial bones may be considered since X-rays of these sites have been found to detect PDB in >90% of patients.^5^ The main purpose of performing imaging is to identify which bones are affected to help decide if a patient’s symptoms might be due to PDB or another cause. Patients who present with imaging suggestive of PDB should be asked if they have pain or discomfort localised to the affected site. The key investigation is ALP, which gives an indication if PDB is metabolically active, but liver function tests, calcium, phosphate, vitamin D, and creatinine are helpful in the differential diagnosis from other conditions such as renal osteodystrophy, primary hyperparathyroidism, and osteomalacia (Table 1). If the patient presents with pain, both imaging and biochemistry should be performed to determine if there is evidence of metabolic activity and to evaluate if the site(s) of the pain coincide with sites of involvement.

**Table 1.** Biochemical features of Paget’s disease of bone and other metabolic bone diseases

**Disease**	**Serum calcium**	**Serum phosphate**	**Serum creatinine**	**Serum PTH**	**Serum 25(OH)D**	**Serum alkaline phosphatase**
PHPT	↑	↓	↔	↑	↔	↔/↑
Osteomalacia	↓	↓	↔	↑	↓↓	↑
Paget’s	↔	↔	↔	↔	↔	↑/↑↑
CKD-MBD	**/↓	↑	↑↑	↑↑	↔	↑
Osteoporosis	↔	↔	↔	↔	↔	↔

CKD-MBD = chronic kidney disease — metabolic bone disease. PHPT = primary hyperparathyroidism. PTH = parathyroid hormone. ** = no change. ↑ = elevated. ↑↑ = markedly elevated. ↓ = reduced. ↔ = within reference range.

## WHEN SHOULD PATIENTS BE REFERRED TO SECONDARY CARE?

Referral to secondary care is advisable in a patient thought to have pain or deformity due to PDB so that further assessment can be performed and treatment offered if appropriate. Referral may not be required in older patients who are asymptomatic where PDB is picked up as an incidental finding because there is no evidence as yet to suggest that anti-Pagetic treatment is of clinical benefit in these circumstances.^1^

## WHAT TREATMENT OPTIONS ARE AVAILABLE AND WHEN SHOULD TREATMENT BE GIVEN?

Bisphosphonates are the treatment of first choice and are indicated in patients with pain localised to an affected site with evidence of increased metabolic activity. Usually this is manifest by an elevation in ALP but when a single bone is affected the ALP may be normal; here a radionuclide bone scan can be useful in assessing if there is local disease activity. In the UK, three bisphosphonates have marketing authorisation for the treatment of PDB (Table 2). Zoledronic acid (ZA) is the treatment of first choice because it is most likely to improve pain and has sustained inhibitory effects on disease activity.^6^ Intravenous pamidronate is also an effective treatment but is now used less commonly. For patients who are not keen on an infusion, oral risedronate represents a good alternative. Calcitonin (100 units three times weekly, by subcutaneous injection) is reserved for the management of bone pain where bisphosphonates are contraindicated because long-term treatment has been associated with an increased risk of certain types of cancer. Although bisphosphonates can improve bone pain significantly in PDB, many patients require additional analgesics to control the elements of pain that may not be due to increased metabolic activity.

**Table 2.** Bisphosphonates licensed for Paget’s disease of bone in the UK

**Drug**	**Route of administration**	**Dose**	**Posology**	**Duration of action**
Risedronate	Oral	30 mg/day	2 months	1–2 years
Zoledronic acid	Intravenous	5 mg	Single infusion	2–5 years
Pamidronate	Intravenous	60 mg	Three infusions	1–2 years

## ORTHOPAEDIC SURGERY

Apart from fracture repair, the most common indication for orthopaedic surgery is arthroplasty for osteoarthritis. Surgery can be technically difficult in PDB due to deformity and the presence of sclerotic bone, but outcomes are generally excellent and having PDB is not a contraindication to referring a patient for joint replacement.^1^

## FUTURE PROSPECTS AND DIRECTIONS

Research is now in progress (ISRCTN11616770) to determine if intervention with bisphosphonates in asymptomatic patients with early disease can halt progression and prevent complications.
